# Diagnosis of Autosomal Dominant Polycystic Kidney Disease in a 66-Year-Old Patient With a Genotype-Phenotype Mismatch

**DOI:** 10.7759/cureus.81717

**Published:** 2025-04-04

**Authors:** Gautam Agrawal, Bhawna Agarwal, Pallavi Shirsat, Kunal Sonavane

**Affiliations:** 1 Nephrology, Independence Health System, Greensburg, USA; 2 Internal Medicine, University of Pittsburgh Medical Center, McKeesport Hospital, McKeesport, USA; 3 Nephrology, Minden Medical Center, Minden, USA; 4 Internal Medicine, Willis Knighton Medical Center, Bossier City, USA

**Keywords:** adpkd patients, asymmetric adpkd, bilateral renal cysts, renal cyst, the oldest patient with adpkd

## Abstract

Autosomal dominant polycystic kidney disease (ADPKD) is a common genetic disorder characterized by the progressive development of renal cysts, ultimately leading to chronic kidney disease (CKD) and end-stage renal disease (ESRD). Patients are typically diagnosed in their 20s or 30s, and the majority have a parent with a known history of the condition. The most common gene mutations associated with ADPKD are *PKD1* and *PKD2*, although other mutations have also been identified. Kidney enlargement rates can vary and serve as a marker for ADPKD progression and the eventual decline in kidney function. The Mayo Imaging Classification (MIC) tool assesses the risk of progression by incorporating height-adjusted total kidney volume (htTKV) and the patient's age. Tolvaptan is recommended for patients at high risk of progression, although it has not been studied in individuals over the age of 65.

This case report focuses on the diagnosis and management of ADPKD in a 66-year-old male with no known family history of the condition. Genetic testing revealed an *IFT140* gene mutation, typically associated with a less severe phenotype. However, the patient was classified as 1C according to the MIC, indicating a high risk of disease progression. This case underscores the challenges of managing severe disease in older patients, given the limited research available for this age group.

## Introduction

Autosomal dominant polycystic kidney disease (ADPKD) is the most common hereditary kidney disease and the fourth leading cause of kidney failure worldwide [1]. The estimated number of individuals in the United States diagnosed with clinically recognized ADPKD ranges from 140,000 to 240,000 [1]. The mean age of ADPKD diagnosis ranges from 27 to 42 years [1]. A known history of ADPKD in one parent is identified in the majority of patients (75%-90%), while a smaller percentage (10%-25%) have no known family history of the disease [2]. ADPKD is characterized by multiple renal cysts, kidney enlargement, and a gradual decline in kidney function. Patients may present with symptoms such as flank pain, hypertension, hematuria, urinary tract infections, kidney stones, and impaired kidney function [3].

The genes most commonly associated with ADPKD are *PKD1* and *PKD2*, with rarer associations including *ALG5*, *ALG9*, *DNAJB11*, *GANAB*, and *IFT140* [1,4]. Pathogenic variants in the *IFT140* gene account for 1%-2% of ADPKD cases [4]. Mutations in the *IFT140* gene are typically associated with a milder phenotype and a slower rate of disease progression to end-stage renal disease (ESRD) [1,5]. The Mayo Imaging Classification (MIC) uses height-adjusted total kidney volume (htTKV) and age to predict the risk of disease progression [6]. Management of ADPKD focuses on addressing clinical symptoms, optimizing blood pressure, and reducing the risk of progression to ESRD.

We present a case of a 66-year-old male patient with no known family history of ADPKD, diagnosed through magnetic resonance imaging (MRI) and found to have an *IFT140* mutation on genetic testing, classified as MIC class 1C.

## Case presentation

We present the case of a 66-year-old male patient who was followed in the nephrology clinic for the management of chronic kidney disease (CKD). His relevant medical history included hypertension, hyperlipidemia, gastroesophageal reflux disease, benign prostatic hyperplasia, and fatty liver. He had no family history of polycystic kidney disease or any known chronic kidney disease.

The patient had a history of knee pain, for which he was taking large doses of ibuprofen for symptom relief. His baseline creatinine level was 1 mg/dL but was found to be elevated to 1.5 mg/dL with an estimated glomerular filtration rate (eGFR) of 46 mL/minute. The physical examination was unremarkable, although he was hypertensive, with a blood pressure of 154/83 mmHg. He was 5 feet 10 inches tall and weighed 249 lbs. His lungs were clear, and his heart rate was normal, with a regular rhythm. He appeared euvolemic, with no peripheral edema. Blood work revealed elevated serum blood urea nitrogen (BUN) and creatinine levels, as shown in Table 1. Other laboratory tests were unremarkable, and his urinalysis was clear, with no protein, white blood cells, or red blood cells.

**Table 1:** Laboratory values BUN: blood urea nitrogen

Laboratory tests	Results	Reference range
Creatinine	1.5 mg/dL	0.7-1.3 mg/dL
BUN	29 mg/dL	7-25 mg/dL
Albumin	4.3 g/dL	3.5-5.7 g/dL
Sodium	138 mEq/L	136-145 mEq/L
Potassium	4.5 mEq/L	3.4-4.5 mEq/L
Calcium	9.7 mg/dL	8.6-10.3 mg/dL
Phosphorus	3.1 mg/dL	2.5-5.0 mg/dL
Hemoglobin	14.8 g/dL	12.7-17.5 g/dL

**Figure 1:**
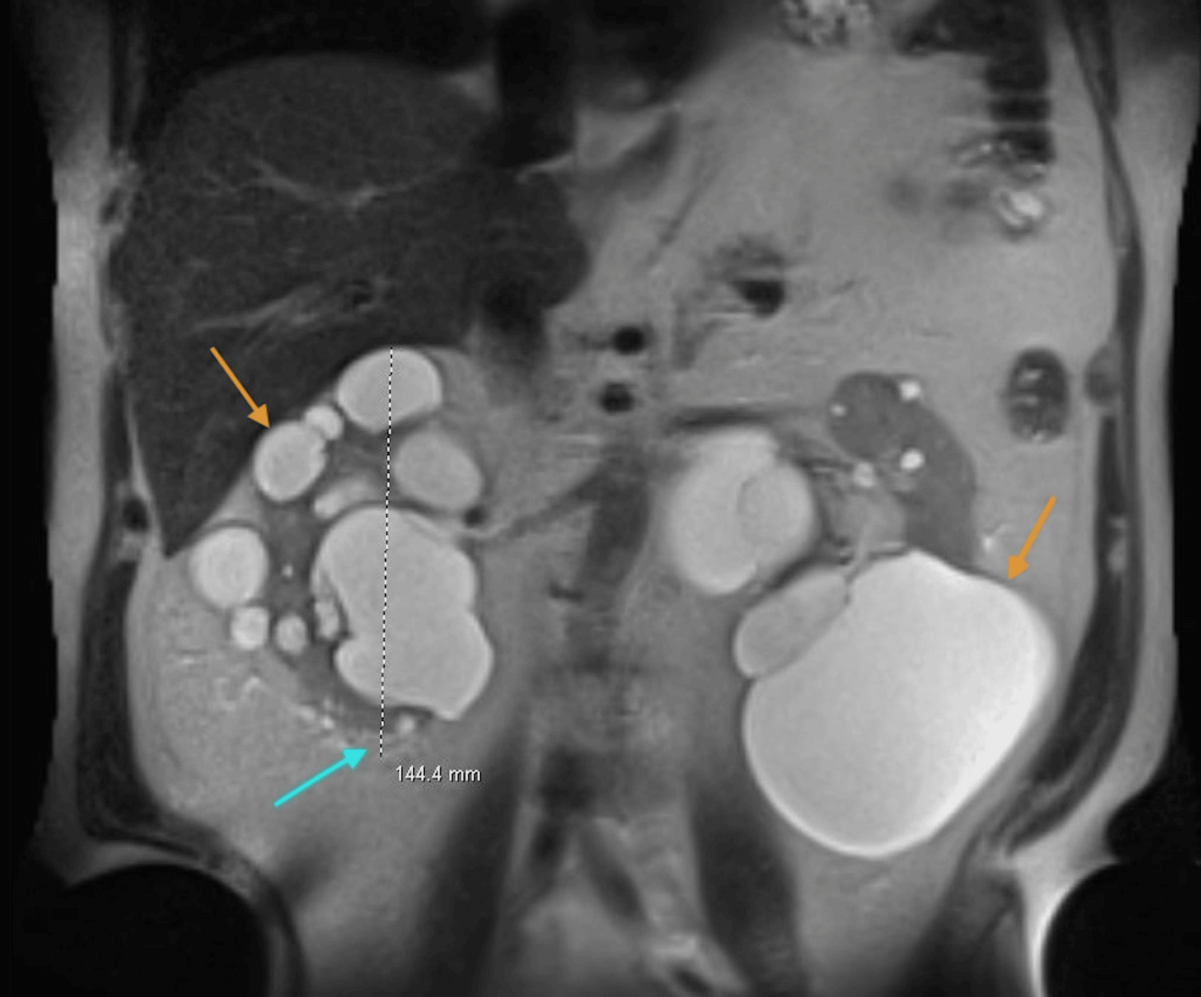
MRI images showing bilateral enlarged kidneys with multiple renal cysts Orange arrows indicate renal cysts. The blue arrow indicates the right kidney coronal length measurements. MRI: magnetic resonance imaging

**Figure 2:**
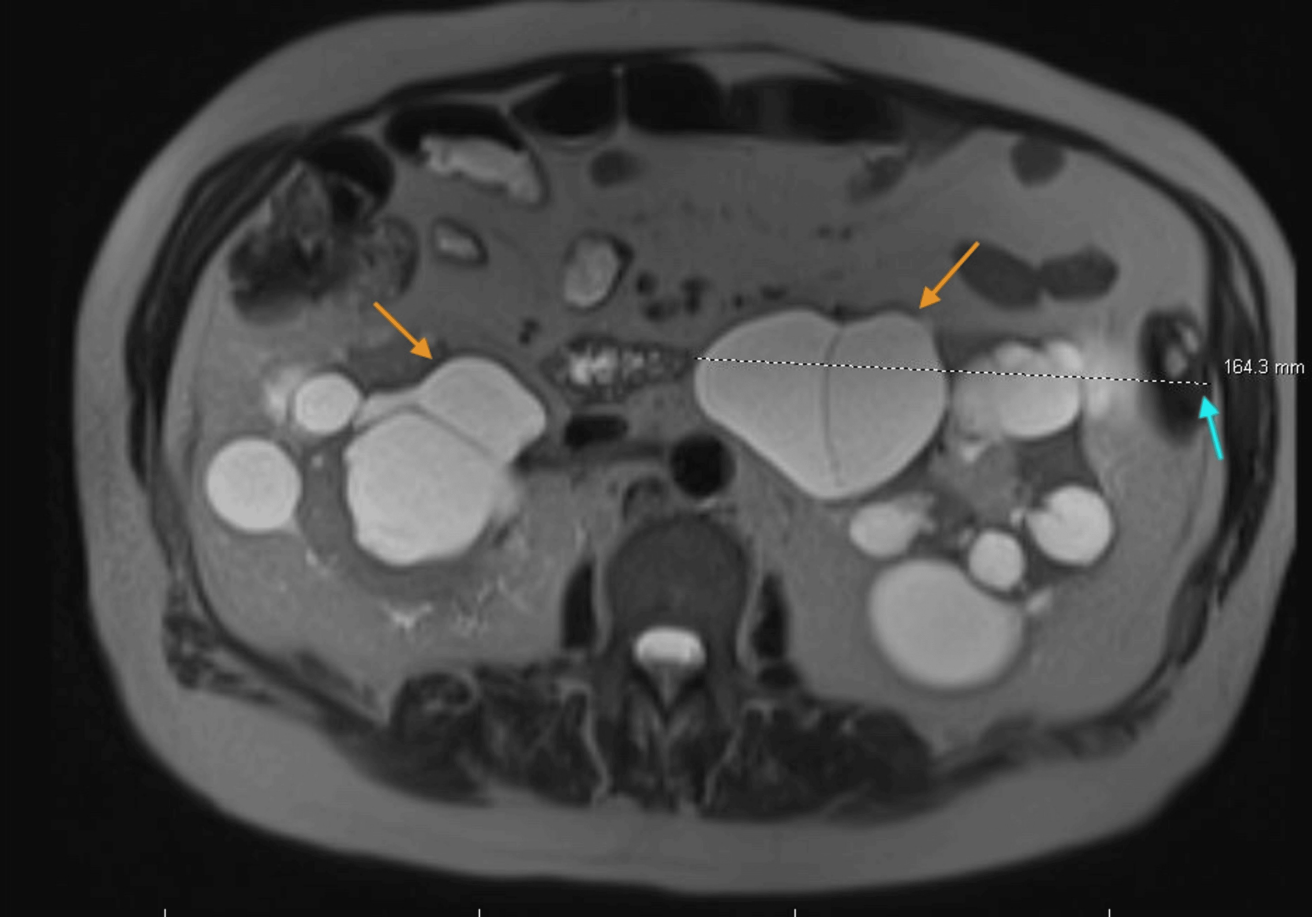
MRI images showing bilateral enlarged kidneys with multiple renal cysts Orange arrows indicate renal cysts. The blue arrow indicates the left kidney width measurement. MRI: magnetic resonance imaging

An ultrasound of his kidneys revealed multiple bilateral cysts. He later underwent an MRI scan, which confirmed the presence of multiple bilateral cysts (22 cysts in the right kidney and 33 in the left kidney), consistent with the diagnosis of polycystic kidney disease. The maximum coronal dimensions of his kidneys were as follows: right kidney: length, 14.4 cm; width, 11.2 cm; and depth, 11.8 cm, and left kidney: length, 20 cm; width, 16.4 cm; and depth, 15.4 cm. MRI images of the patient, shown in Figure 1 and Figure 2, display severely enlarged kidneys with multiple bilateral renal cysts.

His total kidney volume (TKV) is calculated using the ellipsoid volume formula: 𝑉 = 𝜋 / 6 × (𝐿 × 𝑊 × 𝐷), where L is the length, W is the width, and D is the depth. Using the above formula, his total kidney volume (TKV) was 3641.3 mL/m. His height-adjusted TKV was 2045.7 mL/m, which falls under the class IC classification of the Mayo Imaging Classification (MIC) for autosomal dominant polycystic kidney disease (ADPKD). Class IC indicates an estimated kidney growth rate of 3%-4.5% per year, which is considered high risk for a decline in the estimated glomerular filtration rate (eGFR).

He underwent genetic testing, which identified an *IFT140* mutation, which was likely pathogenic, typically associated with mild polycystic kidney disease. However, in this patient, the disease was more severe, as indicated by the Mayo Imaging Classification. After shared decision-making, tolvaptan was discussed, but we decided not to start as it has not been studied in his age group and has potential side effects, including polyuria and liver dysfunction. He was advised to stop and avoid all non-steroidal anti-inflammatory drugs (NSAIDs) and was recommended to increase his hydration to more than 3 L per day, along with intensive blood pressure control.

## Discussion

Our case highlights an elderly male patient with autosomal dominant polycystic kidney disease (ADPKD) who has no known family history, presenting with a genotype-phenotype mismatch and challenges in disease management due to his age. The *IFT140* gene mutation is typically associated with a milder form of the disease and a lower risk of renal function decline [5]. However, our patient was found to be at high risk of disease progression. MIC class 1C-1E has been associated with a higher likelihood of progression to renal failure [1], and our patient was classified as MIC class 1C, further emphasizing the genotype-phenotype mismatch. A similar case was reported of a young female with ADPKD and MIC class 1E, showing high disease progression risk despite a *GANAB* gene mutation, typically linked to milder ADPKD [7].

Tolvaptan is currently the only available treatment to slow disease progression in ADPKD. It is a selective vasopressin V2 receptor antagonist that has been shown to slow the progression of renal disease in patients with ADPKD. It works by reducing cAMP-mediated cyst proliferation. The common side effects associated with tolvaptan include polyuria, polydipsia, hypernatremia, and elevated liver function tests. Two landmark trials (Tolvaptan Efficacy and Safety in the Management of ADPKD and Its Outcomes (TEMPO 3:4) [8] and Replicating Evidence of Preserved Renal Function: An Investigation of Tolvaptan Safety and Efficacy in ADPKD (REPRISE) [9]) have demonstrated that tolvaptan reduces the risk of progression and slows the decline of GFR. TEMPO 3:4 enrolled patients aged 18-50 years [8], while REPRISE included patients aged 18-55 years with an estimated GFR (eGFR) of 25-65 mL/minute/1.73 m² or those aged 56-65 years with an eGFR of 25-44 mL/minute/1.73 m², with patients aged 56-65 years comprising less than 15% of the study population [9]. Notably, tolvaptan has not been studied in patients over 65 years of age. Therefore, shared decision-making is recommended for patients over 55 years old who are at high risk of disease progression, given the limited clinical data available for this population [10]. All patients with ADPKD are recommended to maintain adequate hydration, follow a low-sodium diet, adopt lifestyle changes to optimize weight, and intensify blood pressure control.

This case highlights the complexities of managing ADPKD in elderly patients with a high risk of disease progression. It is crucial to evaluate ADPKD patients thoroughly and optimize treatment strategies to slow the decline in estimated glomerular filtration rate (eGFR), particularly for those at high risk for progression of renal failure.

## Conclusions

ADPKD is a genetic disorder commonly associated with a family history of the condition, with most patients being diagnosed between the ages of 27 and 42. However, our 66-year-old patient had no known family history of ADPKD and was found to have a genotype-phenotype mismatch. Managing ADPKD in elderly patients is particularly challenging due to the limited number of studies focused on this population. Tolvaptan, the only approved medication for reducing the risk of disease progression in ADPKD, has not been studied in patients over 65 years of age. In individuals over 55, shared decision-making is recommended when considering tolvaptan therapy. Further research is necessary to optimize the management of ADPKD in elderly patients and to assess the risks and benefits of tolvaptan in this age group.
